# Modified Recombinant Proteins Can Be Exported via the Sec Pathway in *Escherichia coli*

**DOI:** 10.1371/journal.pone.0042519

**Published:** 2012-08-13

**Authors:** Nan Chen, Fu-Lin Hong, Hai-Hong Wang, Qi-Hang Yuan, Wan-Yan Ma, Xu-Na Gao, Rui Shi, Rui-Juan Zhang, Chang-Sheng Sun, Sheng-Bin Wang

**Affiliations:** College of Life Sciences, South China Agricultural University, Guangzhou, Guangdong, P. R. China; University of Kent, United Kingdom

## Abstract

The correct folding of a protein is a pre-requirement for its proper posttranslational modification. The *Escherichia coli* Sec pathway, in which preproteins, in an unfolded, translocation-competent state, are rapidly secreted across the cytoplasmic membrane, is commonly assumed to be unfavorable for their modification in the cytosol. Whether posttranslationally modified recombinant preproteins can be efficiently transported via the Sec pathway, however, remains unclear. ACP and BCCP domain (BCCP87) are carrier proteins that can be converted into active phosphopantetheinylated ACP (holo-ACP) and biotinylated-BCCP (holo-BCCP) by AcpS and BirA, respectively. In the present study, we show that, when ACP or BCCP87 is fused to the C-terminus of secretory protein YebF or MBP, the resulting fusion protein preYebF-ACP, preYebF-BCCP87, preMBP-ACP or preMBP-BCCP87 can be modified and then secreted. Our data demonstrate that posttranslational modification of preYebF-ACP, preYebF-BCCP87 preMBP-ACP and preMBP-BCCP87 can take place in the cytosol prior to translocation, and the Sec machinery accommodates these previously modified fusion proteins. High levels of active holo-ACP and holo-BCCP87 are achieved when AcpS or BirA is co-expressed, especially when sodium azide is used to retard their translocation across the inner membrane. Our results also provide an alternative to achieve a high level of modified recombinant proteins expressed extracellularly.

## Introduction

Posttranslational modifications play important roles in the regulation of protein activities in all known living organisms. Representative posttranslational modifications include phosphorylation, acetylation, ubiquitination, sumoylation and glycosylation [1]. It is generally presumed that *Escherichia coli* are unable to exert posttranslational modification of recombinant proteins. However, several approaches have been developed to produce recombinant proteins with posttranslational modifications. One commonly used method is to co-express a target protein together with the corresponding modification enzyme in *E. coli*, such as enzymes that catalyze phosphorylation [2], acetylation [3], SUMO-modification [4], phosphopantetheinylation [5] and biotinylation [6].

There are two major protein targeting routes in *E. coli* general Sec system that directs preproteins to the Sec translocase. Most preproteins are targeted posttranslationally via the secretion-dedicated molecular chaperone SecB. In this pathway, the preproteins are released from the ribosome after their synthesis and guided to the Sec-translocase by SecB, which maintains them in a translocation-competent, unfolded state, then promoted through the SecYEG pore in cytoplasmic membrane by SecA, a molecular motor dependent on ATP hydrolysis [7], [8], [9]. Since it is generally believed that translocation-competent preproteins must be kept in an unfolded SecB-bound state, which is unfavorable for the recognition and modification by the corresponding enzymes in the cytosol, whether recombinant protein precursors can be posttranslationally modified and subsequently accommodated by the Sec machinery is still unclear.

The secretion of target proteins into culture medium has several advantages, such as an easier downstream processing and better compatibility with continuous culturing [10]. It is generally assumed that laboratory *E. coli* strains rarely secrete proteins into the surrounding medium. Great efforts have been made to enhance the extracellular production of target proteins, such as promotion of translocation, improvement of periplasmic release, and protection of target proteins from degradation [11]. Several approaches have been developed successfully to achieve extracellular production of recombinant proteins [11], [12], [13]. One of the representative examples is to make use of the carrier proteins [14]. YebF, a secreted peptide, was recently demonstrated to be an effective fusion partner that carried recombinant target proteins from the cytosol of *E. coli* into the medium [15]. However, modification of secretory recombinant proteins has been less characterized.

Phosphopantetheinylation and biotinylation are two posttranslational modifications, which occur widely in living organisms, especially in metabolic enzymes to regulate their activity, such as aromatic carboxylic acid reductase, non-ribosomal peptide synthetases (NRPSs), polyketide synthases (PKSs) and carboxylases [16], [17], [18]. Biotinylation of histone H4 has been recently found to be involved in the regulation of nucleosome structure and gene transcription [19]. Biotinylation is also regarded as a useful protein tag in a variety of biochemical experiments [20], [21]. In the present study, acyl carrier protein (ACP) and biotin carboxyl carrier protein domain (BCCP87) [22] are fused with secretory protein YebF or MBP, respectively, and expressed in *E. coli*. We further investigate the posttranslational modification of the resulting fusion proteins in the cytosol and their subsequent secretion process in *E. coli*. Our data indicate that recombinant protein precursors can be effectively modified in the cytosol prior to translocation, and the Sec machinery accommodates these previously modified preproteins. Our results also provide an alternative strategy for the extracellular production of modified recombinant proteins.

## Results

### Extracellular expression of fusion protein preYebF-ACP and preYebF-BCCP87 in *E. coli*

**Figure 1:**
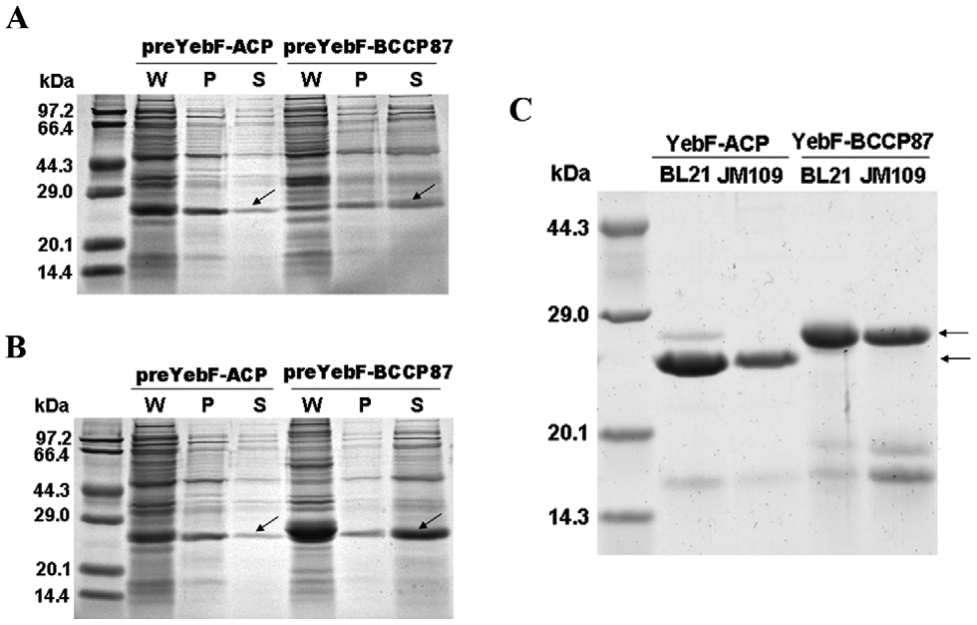
SDS-PAGE analysis of the expression and purification of recombinant fusion proteins. After induction with IPTG for 12 h, *E. coli* cells were harvested, total cell contents (W) were analyzed together with supernatant (S) and periplasm samples (P) after centrifugation and osmotic shock treatment. *Panel A*: fusion proteins were expressed in *E. coli* strain JM109. *Panel B*: fusion proteins were expressed in *E. coli* strain BL21 (DE3). *Panel C*: fusion proteins expressed extracellularly for 12 h were purified by one-step Ni-chelating affinity chromatography.

Extracellular production of recombinant proteins has several advantages, especially for easier downstream purification of target proteins. To achieve extracellular expression of ACP and BCCP87, YebF was selected as the fusion partner, which is transported from the cytoplasm to the periplasm by the Sec-dependent system in the inner membrane, and subsequently exported to the culture medium by an undefined mechanism [15]. Fusion protein preYebF-ACP and preYebF-BCCP87 were constructed, in which a His-tag was inserted between YebF signal peptide and its mature form to facilitate the downstream purification procedure, and a site-specific protease TEV recognition site was also added at the N-terminus of ACP and BCCP87 respectively to render specific cleavage of fusion proteins by protease TEV [23] (Figure S1). As shown in Figure 1A and Figure 1B, in the two *E. coli* strains tested, both YebF-ACP and YebF-BCCP87 were successfully exported across the inner and outer membrane, and accumulated in the culture medium. Compared with the expression of preYeb-ACP and preYeb-BCCP87, fusion proteins without the signal peptide accumulated in cytosol (Figure S2). Recombinant fusion proteins could be recovered to high purity by one-step Ni-chelating chromatography (Figure 1C).

### Modification of secretory fusion proteins

**Figure 2:**
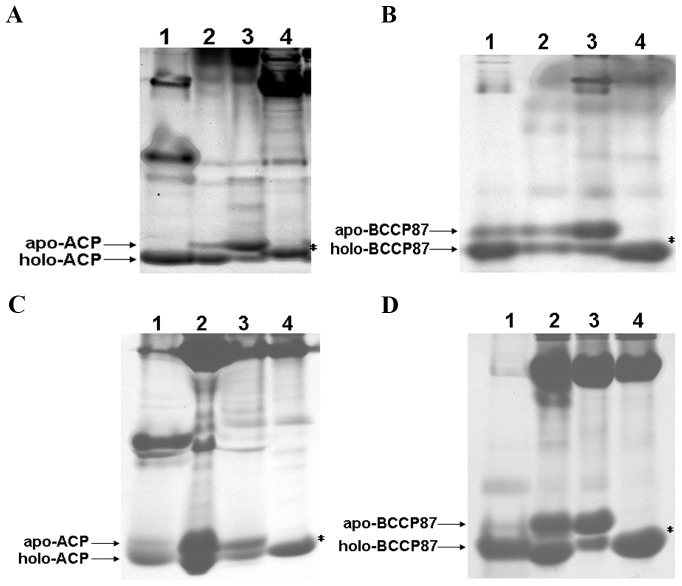
Non-denaturing urea-PAGE analysis of ACP and BCCP87 pool compositions. Fusion proteins were purified by Ni-chelating affinity chromatography, treated with protease TEV, and then subjected to urea-PAGE analysis. *Panel A*: ACP was fused to C-terminus of YebF, lane 1, ACP modified with AcpS *in vitro* as standard; lane 2, YebF-ACP expressed in cytosol; lane 3, YebF-ACP expressed extracellularly; lane 4, YebF-mACP expressed extracellularly. *Panel B*: BCCP87 was fused to C-terminus of YebF, lane 1, BCCP87 modified with BirA *in vitro* as standard; lane 2, YebF-BCCP87 expressed in cytosol; lane 3, YebF-BCCP87 expressed extracellularly; lane 4, YebF-mBCCP87 expressed extracellularly. *Panel C*: ACP was fused to C-terminus of MBP, lane 1, ACP modified with AcpS *in vitro* as standard; lane 2, MBP-ACP expressed in cytosol; lane 3, MBP-ACP accumulated in periplasm; lane 4, MBP-mACP accumulated in periplasm. *Panel D*: BCCP87 was fused to C-terminus of MBP, lane 1, BCCP87 modified with BirA *in vitro* as standard; lane 2, MBP-BCCP87 expressed in cytosol; lane 3, MBP-BCCP87 accumulated in periplasm; lane 4, MBP-mBCCP87 accumulated in periplasm.* indicates the mutant ACP (mACP) or mutant BCCP87 (mBCCP87).

The correct recognition of a substrate by its corresponding enzyme requires it to fold into a defined conformation. In the posttranslational Sec-dependent pathway, preprotein synthesis and translocation are uncoupled events. Preproteins are kept in unfolded states by the secretion-dedicated chaperone SecB before translocation [7], [8]. Whether the recombinant protein precursors can be recognized, modified by their corresponding enzymes in the cytosol, and subsequently admitted by Sec translocases are still unclear. In the cytosol of *E. coli*, ACP is posttranslationally modified by holo-(acyl carrier protein) synthase (AcpS), and converted to active phosphopantetheinylated ACP (holo-ACP) [24]; similarly, BCCP is converted to active biotinylated BCCP (holo-BCCP) by biotin ligase enzyme (BirA) [25]. Additionally, the apo form of ACP or BCCP87 could be clearly resolved from their holo form by the conformationally sensitive PAGE assay (urea-PAGE) based on their differences in molecular size, structural stability and charges [26], [27], [28], [29]. We purified fusion proteins accumulated in culture medium by Ni-chelating affinity chromatography, treated them with site-specific protease TEV to release protein ACP and BCCP87, which in turn were subjected to urea-PAGE analysis. Interestingly, phosphopantetheinylated ACP and biotinylated BCCP87 were detected in the urea-PAGE gel (Figure 2A and 2B). In these figures, we also added mutant ACP (S36A) and BCCP87 (K122H) as useful controls, in which their modification sites were mutated and could not be modified *in vivo*. As expected, these mutants presented only one conformational form. These data provided preliminary evidence that fusion protein precursors could be modified in the cytosol, and subsequently secreted via the Sec system.

**Figure 3:**
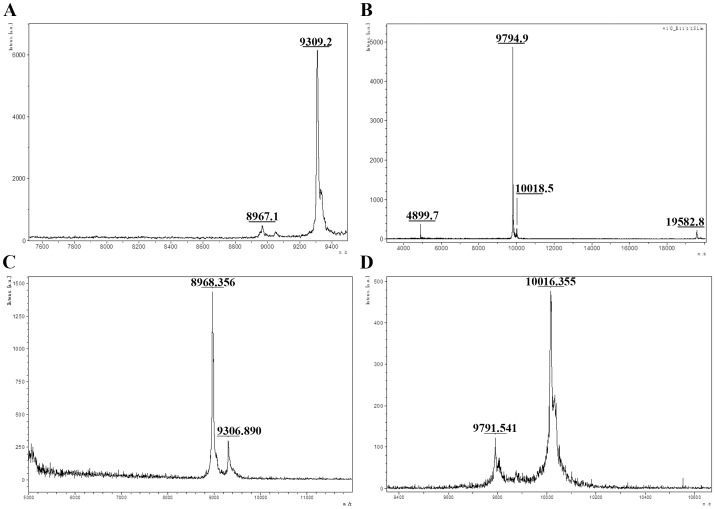
MALDI-TOF mass spectral analysis of recombinant ACP and BCCP87. Fusion proteins were purified by Ni-chelating affinity chromatography, treated with specific protease TEV, and then subjected to MALDI-TOF mass spectral analysis. The calculated molecular weight (MW) of recombinant apo-BCCP87 is 9791 Da, holo-BCCP87 is 10017 Da, apo-ACP is 8939 Da, holo-ACP is 9279 Da. The difference in molecular weight between apo-BCCP87 and holo-BCCP87 is 226 Da, between apo-ACP and holo-ACP is 340 Da. *Panel A*: MALDI-TOF mass spectrum of ACP resulted from the expression product of *preMBP-ACP*. *Panel B*: MALDI-TOF mass spectrum of BCCP87 resulted from the expression product of *preMBP-BCCP87*. *Panel C*: MALDI-TOF mass spectrum of ACP resulted from the expression product of *preYebF-ACP*, which was co-expressed with modification enzyme *AcpS*. *Panel D*: MALDI-TOF mass spectrum of BCCP87 resulted from the expression product of *preYebF-BCCP87*, which was co-expressed with modification enzyme *BirA*.

The precursor of maltose binding protein (MBP) is a native substrate of the posttranslational Sec-dependent pathway, and its translocation strictly depends on the molecular chaperone SecB [30]. To further investigate the modification of preproteins in cytosol and their subsequent translocation, we constructed another set of fusion proteins, preMBP-ACP and preMBP-BCCP87 (Figure S1). Recombinant fusion protein MBP-ACP and MBP-BCCP87 were extracted from periplasm, purified by Ni-chelating affinity chromatography, treated with site-specific protease TEV, and subjected to urea-PAGE analysis. Modified forms of both ACP and BCCP87 were detected in the urea-PAGE gel (Figure 2C and 2D). To further confirm the existence of biotinylated BCCP87 and phosphopantetheinylated ACP, periplasmic fusion protein MBP-ACP and MBP-BCCP87 treated with protease TEV were also subjected to MALDI-TOF-MS analysis. As shown in Figure 3A and 3B, the peak with mass 9309.2 corresponded to the molecular weight of phosphopantetheinylated ACP (holo form), and the peak of molecular mass 10018.5 corresponded to the biotinylated BCCP87 (holo form). We also analyzed the ACP pools and BCCP87 pools extracellularly expressed using MALDI-TOF-MS. Figure 3C and 3D clearly showed the existence of modified ACP and BCCP87.

**Figure 4:**
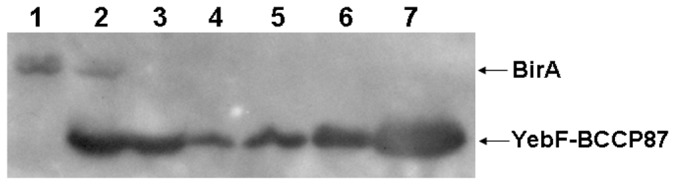
Investigation of the integrity of *E. coli* cells. Cells harboring pSY-B and pBAD34-B for coexpression of preYebF-BCCP87 together with cytosolic His_6_-BirA were used in lane 2, 4, 5, 6, 7; cells harboring pSY-B for the expression of preYebF-BCCP87 were used in lane 3. Compartmentation of recombinant protein His_6_-BirA and His_6_-YebF-BCCP87 were analyzed by immunoblotting with antibody against His-tag. Lane 1, purified recombinant His_6_-BirA; lane 2 and lane 3, whole cell lysate; lane 4, periplasmic fraction; lane 5, supernatants of cultures induced for 12 h; lane 6, supernatants of cultures induced for 24 h; lane 7, supernatants of cultures concentrated to 30-fold after induction for 24 h.

As for fusion protein YebF-ACP and YebF-BCCP87, it is possible that the modification of these fusion proteins was brought about in the culture medium by corresponding enzymes released from lysed host cells. To probe the possibility of this phenomenon, we co-expressed preYebF-BCCP87 together with His-tagged BirA, and investigated the compartmentation of recombinant BirA by immunoblotting. As shown in Figure 4, under our culture conditions, there is no detectable modification enzyme BirA both in the periplasm and culture medium. These results showed that the amount of cytosolic modification enzymes released from lysed cells or from membrane leakage was too small to be probed, and it is almost impossible for fusion proteins to be modified beyond the cytosol.

Our data demonstrated that recombinant protein precursors could be modified by cytosolic corresponding enzymes prior to translocation, and Sec translocases accommodate these previously modified substrates.

### Achievement of high degree of modification of fusion proteins by co-expression of modification enzymes

**Figure 5:**
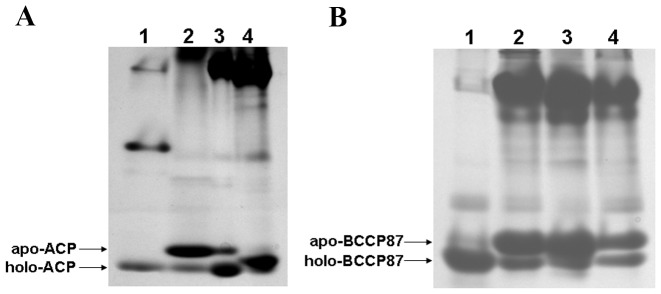
Effect of co-expression of modification enzymes and addition of sodium azide in culture medium on posttranslational modification of ACP and BCCP87. Purified recombinant fusion proteins expressed extracellularly were treated with protease TEV, and then subjected to urea-PAGE analysis. *Panel A*: Lane 1, ACP modified with AcpS *in vitro* as standard; Lane 2, YebF-ACP expressed extracellularly; Lane 3, YebF-ACP expressed extracellularly and coexpressed with cytosolic modification enzyme AcpS; Lane 4, YebF-ACP expressed extracellularly and coexpressed with cytosolic modification enzyme AcpS, and with addition of 1 mM sodium azide in culture medium. *Panel B*: Lane 1, BCCP87 modified with BirA *in vitro* as standard; Lane 2, YebF-BCCP87 expressed extracellularly; Lane 3, YebF-BCCP87 expressed extracellularly and coexpressed with cytosolic modification enzyme BirA; Lane 4, YebF-BCCP87 expressed extracellularly and coexpressed with cytosolic modification enzyme BirA, and with addition of 1 mM sodium azide in culture medium.

Although phosphopantetheinylated YebF-ACP and biotinylated YebF-BCCP87 could be detected in the culture medium, the modified proteins only accounted for a fraction of the exported target proteins. It was demonstrated recently that biotinylation is controlled by the rate of enzyme-substrate association [31]. We therefore speculated that such less efficient modification resulted from a low level of endogenous modification enzymes or rapid exportation of preproteins. In cytoplasm, apo form of ACP and BCCP87 are converted to their active holo forms by AcpS and BirA, respectively [24], [25]. To achieve high degree of modification of fusion protein, preYebF-ACP together with cytosolic AcpS, and preYebF-BCCP87 together with cytosolic BirA were co-expressed. As expected for ACP and BCCP87, the ratio of holo to apo form was significantly increased in this co-expression system (Figure 5A and 5B).

In the Sec-dependent posttranslational pathway, translocation of preproteins across cytoplasmic membrane is driven by the ATP-dependent motor protein SecA [7]. We postulated that temporal accumulation of fusion protein precursors in cytosol may help them to interact with the corresponding modification enzymes, which in turn contributed to the higher degree of modification of fusion proteins. Sodium azide has been found to be a specific inhibitor of the ATPase activity of motor protein SecA [32]. As shown in Figure 5A and 5B, the highest degree of modification of ACP and BCCP87 were obtained by the addition of 1 mM sodium azide into the culture medium to retard the translocation of fusion protein precursors.

**Figure 6:**
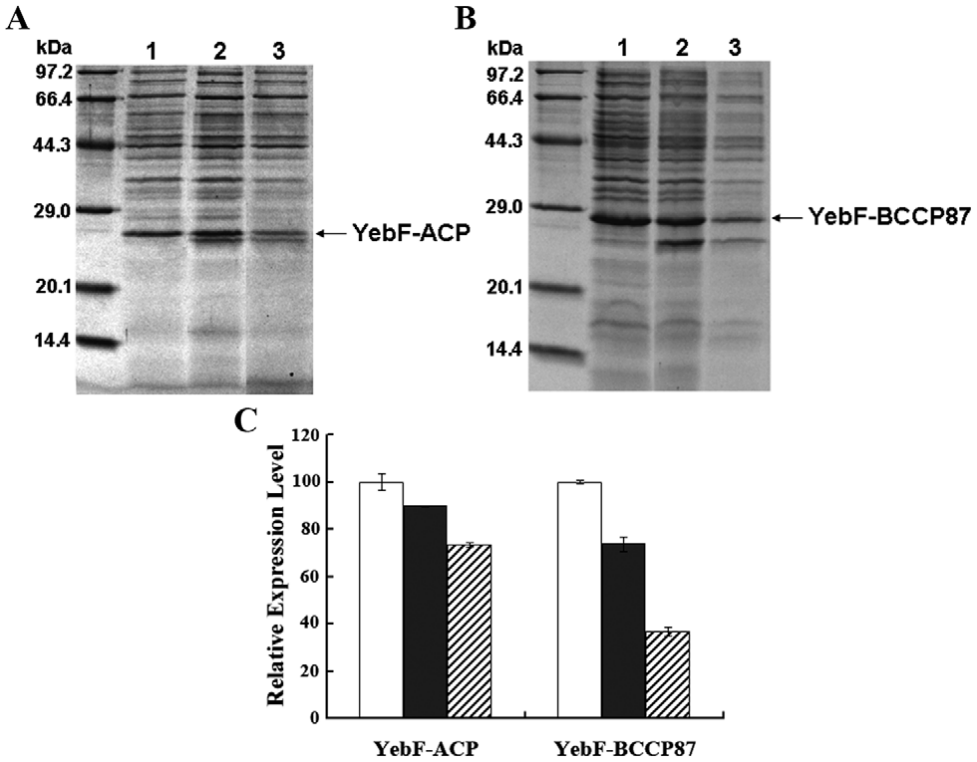
Effect of co-expression of modification enzymes and addition of sodium azide in culture medium on the expression level of fusion proteins. After induction with IPTG for 24 h, *E. coli* cells were removed by centrifugation, supernatants were analyzed with SDS-PAGE (*Panel A* and *Panel B*). *Panel A*: Lane 1, YebF-ACP expressed extracellularly; Lane 2, YebF-ACP expressed extracellularly and coexpressed with cytosolic modification enzyme AcpS; Lane 3, YebF-ACP expressed extracellularly and coexpressed with cytosolic modification enzyme AcpS, and with addition of 1 mM sodium azide in culture medium. *Panel B*: Lane 1, YebF-BCCP87 expressed extracellularly; Lane 2, YebF-BCCP87 expressed extracellularly and coexpressed with cytosolic modification enzyme BirA; Lane 3, YebF-BCCP87 expressed extracellularly and coexpressed with cytosolic modification enzyme BirA, and with addition of 1 mM sodium azide in culture medium. *Panel C*, Target proteins was quantified by Quantity Tools (Image Lab™ software, Version 2.0, BIO-RAD). The densitometric value of the band of YebF-ACP or YebF-BCCP87 expressed extracellularly was set to be 1. Each bar represented the mean ± standard error, three replicates. Open bars: fusion proteins expressed extracellularly; Filled bars: fusion proteins expressed extracellularly and coexpressed with corresponding cytosolic modification enzymes; Hatched bars: fusion proteins expressed extracellularly and coexpressed with corresponding cytosolic modification enzymes, and with addition of 1 mM sodium azide in culture medium.

We also investigated the potential effects of co-expression of target proteins together with corresponding enzymes and addition of inhibitor sodium azide on the extracellular expression level of fusion proteins. Co-expression of preYebF-ACP together with AcpS, and preYebF-BCCP87 with BirA resulted in an approximate 16% decrease in fusion protein level, while addition of sodium azide into the culture medium led to an approximate 55% decrease (Figure 6).

## Discussion

It is generally assumed that, in the posttranslational Sec-dependent pathway, preproteins are kept in an unfolded translocation-competent state in association with SecB [7]. However, *in vitro* data have demonstrated recently that tightly folded human cardiac Ig-like domain I27 fused to the C terminus of preOmpA could be transported effectively by Sec-translocases [33]. It has also been reported that the biotin-accepting domain fused to the carboxyl terminus of periplasmic protein alkaline phosphatase or TEM β-lactamase, could be biotinylated in the cytosol, and then exported by *E. coli* [34]. Previous results suggest that preproteins containing a folded domain may be amenable to Sec machinery. In the posttranslational Sec-dependent pathway, translation and translocation are uncoupled events, which should leave the folded domain contained in preproteins to interact with cytosolic corresponding enzymes, and to be modified. Based on this hypothesis, we chose two fusion partners to construct secretory chimeras, and investigated their modification. One was preYebF, which has recently been taken as an effective carrier protein, and is exported in a two-step process, from cytoplasm to periplasm by the Sec dependent system, and from periplasm to the medium by an undefined mechanism [15]. The other was preMBP, a model substrate of the Sec-dependent pathway [30]. Our SDS-PAGE analysis showed that these secretory fusion proteins could be effectively transported across the inner membrane, and of these proteins, YebF-ACP and YebF-BCCP87 were further exported across the outer membrane into the culture medium. We first analyzed the ACP and BCCP87 pools accumulated in the culture medium with both conformationally sensitive urea-PAGE and mass spectrometry. As expected, modified YebF-ACP and YebF-BCCP87 were both detected. We further investigated the modification of MBP-ACP and MBP-BCCP87 accumulated in the periplasm. Similarly, the occurrence of their modification was also confirmed by both urea-PAGE and mass spectrometry.

Modified target proteins accumulated in culture medium may result from the lysis of a small fraction of host cells. To exclude this possibility, we co-expressed His-tagged BirA with preYeb-BCCP87 and examined their compartmentation by immunoblotting. His-tagged BirA expressed in cytosol could not be detected both in the periplasm and culture medium, while Yeb-BCCP87 was obviously detectable, indicating that it was almost impossible for target proteins to be modified beyond the cytosol. Our evidence clearly demonstrated that ACP or BCCP87 contained in fusion protein precursors was first modified in the cytosol, and then exported. *E. coli* Sec machinery could accommodate these previously folded proteins contained in fusion protein precursors.

Kinetic analysis has indicated that interactions between enzyme and substrate control the posttranslational biotinylation [31]. When modification of target proteins contained in fusion protein precursors takes place in the cytosol prior to translocation, the amount of cytosolic corresponding enzymes should affect this process. We co-expressed preYebF-ACP with cytosolic AcpS, and preYebF-BCCP87 with cytosolic BirA, respectively. As shown in Figure 5, higher degree of modification of ACP and BCCP was achieved with the increased modification enzyme levels in cytosol.

The rapid kinetics of protein export may affect interactions between protein precursors and cytosolic enzymes, leading to the inefficient modification of preproteins prior to translocation [34]. As shown in Figure 2, under normal culture conditions, only a small fraction of the exported ACP or BCCP87 was modified, whenever they were fused with YebF or MBP. Retarded translocation of recombinant protein precursors should provide them with more reaction time to interact with the corresponding modification enzymes, leading to higher degree of modification of target proteins. Our results clearly demonstrated that the highest degree of modification of fusion proteins was achieved when fusion protein precursors were co-expressed with their corresponding cytosolic modification enzymes, and with addition of 1 mM sodium azide in the culture medium, a specific inhibitor of the ATPase activity of motor protein SecA, to retard the translocation of fusion protein precursors [32]. Our study provides an alternative strategy to achieve high degree of modification of target proteins expressed extracellularly.

Tat pathway is another potential system for secretory production of modified recombinant proteins, since it catalyses the translocation of secretory proteins in their folded state [8]. However, the application of this system for this purpose has been plagued by relatively low translocation efficiencies, especially for large fusion proteins, while the Sec pathway has been the primary vehicle for transporting recombinant proteins to the periplasm in biotechnological applications [35], [36]. The alternative strategy, which we presented here, will make downstream processing of active modified target proteins much easier, and more compatible with continuous culturing.

## Materials and Methods

### Bacterial strains and culture conditions

*E. coli* strain BL21 (DE3) and JM109 were used as hosts for protein expression. *E. coli* strains were grown on LB medium containing kanamycin at 50 mg/L, chloramphenicol at 30 mg/L, and ampicillin at 100 mg/L solely or in combination for transformant selection.

### Construction of expression vectors

All primers designed for vector construction are listed in Table 1. ACP, BCCP87, and BirA genes were amplified from *E. coli* strain MG1655 chromosome, and YebF gene was amplified from *E. coli* strain BL21 (DE3) chromosome. To insert a His_6_-tag between the YebF signal peptide and its mature form, we first amplified *YebF* fragment 1 with primer 1 and primer 2, and *YebF* fragment 2 with primer 3 and primer 4; then a hybrid *YebF* was synthesized by overlap extension PCR with two *YebF* fragments [37], digested by *Noc*I and *Bam*HI, and inserted into pET28b, generating vector pSY.

**Table 1:** Primers designed for vector construction and site-directed mutagenesis.

No.	Length	Oligo Sequences (5′→3′)
**1**	30	ATACCATGGGAAAAAGAGGGGCGTTTTTAG
**2**	44	GTGATGATGATGATGATGGCTAGCGAAAACTGATGCGCAGGCAG
**3**	44	AGCCATCATCATCATCATCACGCCAATAATGAAACCAGCAAGTC
**4**	55	TCGGATCCAGCACCACCACCACCAGAAGCCGGACGCCGCTGATATTCCGCCATTC
**5**	39	GGCAGCTTCTGGTGGTGGTGGTGAAGCGCCAGCAGCAGC
**6**	27	TGTAAGCTTACTCGATGACGACCAGCG
**7**	41	GGCAGCTTCTGGTGGTGGTGGTAGCACTATCGAAGAACGCG
**8**	28	GCAAGCTTACGCCTGGTGGCCGTTGATG
**9**	46	GACGAATTCGAAAACCTGTACTTTCAGGCAGCTTCTGGTGGTGGTG
**10**	50	CATCCATGGGCCATCATCATCATCATCACAAGGATAACACCGTGCCACTG
**11**	27	GCAAGCTTATTTTTCTGCACTACGCAG
**12**	43	GTTGAAGACCTGGGCGCGGATGCTCTTGACACCGTTGAGCTGG
**13**	43	CCAGCTCAACGGTGTCAAGAGCATCCGCGCCCAGGTCTTCAAC
**14**	44	CTGTGCATCGTTGAAGCCATGCAC ATGATGAACCAGATCGAAGC
**15**	44	GCTTCGATCTGGTTCATCATGTGCATGGCTTCAACGATGCACAG

To achieve their secretory expression, ACP and BCCP87 were fused to the C-terminus of preYebF or preMBP, respectively. Primer 5 and primer 6 were used first, and then primer 9 and primer 6 to amplify hybrid *BCCP87* encoding BCCP87 with a protease TEV recognition site ENLYFQ at its N-terminus. Similarly hybrid *ACP* encoding ACP with ENLYFQ at its N-terminus was amplified with primer 7 and primer 8, then with primer 9 and primer 8. The resulting amplicons were digested with *Eco*RI and *Hin*dIII, and inserted into pSY, generating vector pSY-A for extracellular expression of preYebF-ACP, and pSY-B for preYebF-BCCP87 in *E. coli* strain BL21 (DE3). Fragment encoding preYebF-ACP or preYebF-BCCP87 was cut from pSY-A or pSY-B, with *Noc*I and *Hin*dIII, and inserted into plasmid pQE2 (QIAGEN) to construct vector pQE2A for extracellular expression of fusion protein preYebF-ACP, and pQE2B for preYebF-BCCP87 in *E. coli* strain JM109. Hybrid *BCCP87* and *ACP* were also inserted into plasmid pSM constructed previously in our laboratory, to construct vector pSM-A for secretory expression of preMBP-ACP, and pSM-B for preMBP-BCCP87 in *E. coli* strain BL21 (DE3).

For their cytosolic expression, hybrid *BCCP87* and *ACP* were inserted into plasmid pCY or pCM, respectively, generating vector pCY-A for the expression of His_6_-YebF-ACP, pCY-B for His_6_-YebF-BCCP87, pCM-A for His_6_-MBP-ACP and pCM-B for His_6_-MBP-BCCP87. All constructs are shown in Figure S1.

*BirA* was amplified with primer 10 and primer 11, digested with *Noc*I and *Hin*dIII, and inserted into pBAD34 to construct vector pBAD34-B for its cytosolic expression. Plasmid pBAD34-A was constructed previously in our laboratory for cytosolic expression of Holo-(acyl carrier protein) synthase AcpS. All constructs was checked by sequencing.

### Protein expression and purification

A single colony of *E. coli* BL21 (DE3) or JM109 cells was used to initiate growth during overnight culture at 37°C in Luria broth (LB) medium supplemented with antibiotics as needed. The overnight culture was then inoculated with fresh LB medium (supplemented with the same antibiotics) at the volume ratio of 1∶100 for BL21 (DE3) or 1∶50 for JM109. Cells incubated to the mid-log phase (absorbance at 600 nm≈0.6) were induced by addition of IPTG to the concentration of 0.1 mM, and incubated for an additional 12 h or 24 h at 30°C for extracellular expression of preYebF-ACP or preYebF-BCCP87; for an additional 4 h at 30°C for secretory expression of preMBP-ACP or preMBP-BCCP87; by addition of IPTG to the concentration of 0.2 mM, and incubated for an additional 4 h at 37°C for cytosolic expression of YebF-ACP, YebF-BCCP87, MBP-ACP and MBP-BCCP87. For co-expression of preYebF-ACP with AcpS, or preYebF-BCCP87 with BirA, cells were induced by addition of 0.1 mM of IPTG and 0.2% arabinose. 10 ml of culture was centrifuged for 5 min at 12000 *g*, the supernatant was kept as the extracellular/medium fraction. Periplasm was prepared by the osmotic shock method, briefly, cells from 10 ml of culture were pelleted, resuspended in 2 ml shock buffer (30 mM Tris-HCl, pH 8.0, 20% sucrose, and 1 mM EDTA), and incubated for 10 min on ice with shaking, after centrifugation at 10000 *g* for 10 min at 4°C, the supernatant was collected. The pellet was resuspended in 2 ml of ice-cold 1 mM MgSO4, incubated on ice for 10 min with vigorous shaking, and centrifuged under the same conditions. Supernatant collected was kept as the periplasmic fraction.

To purify the exported recombinant fusion proteins, the extracellular fraction or periplasmic fraction was directly mixed with 2×buffer A (50 mM NaH_2_PO_4_, 300 mM NaCl, 20 mM imidazole, pH 8.0), and loaded onto a Ni–Sepharose affinity column (GE Healthcare). The loaded column was washed three times with washing buffer (50 mM NaH_2_PO_4_, 300 mM NaCl, 40 mM imidazole, pH 8.0), after which the fusion protein was eluted with elution buffer (50 mM NaH_2_PO_4_, 300 mM NaCl, 500 mM imidazole, pH 8.0). To purify the cytosolic recombinant fusion proteins, 10 ml of culture were harvested by centrifugation at 5000 *g* for 10 min, resuspended in 4 ml buffer A (50 mM NaH_2_PO_4_, 0.3 M NaCl, 20 mM imidazole, pH 8.0), and then lysed by sonication on ice. After sonication, the lysate was centrifuged at 5000 *g* for 15 min to remove cell debris, then the supernatants were recovered by centrifugation at 12000 *g* for 20 min, and subsequently applied to the Ni–Sepharose affinity column (GE Healthcare) followed by the same purification procedure mentioned above.

### Mutation of ACP and BCCP87

In *E. coli*, apo-ACP is modified via transfer of 4′-phosphopantetheine from coenzyme A to its conserved serine 36 γ-OH; the biotin group is attached posttranslationally to a lysine 122 residue in BCCP by the enzyme BirA [24], [38]. To avoid the modification of recombinant ACP and BCCP87 during their expression, we constructed mutant ACP S36A (mACP) and BCCP87 K122H (mBCCP87) using site-specific mutagenesis [39]. Plasmid pSY-A, pSM-A, pSY-B and pSM-B were used as templates, respectively. Primer 12 and primer 13 were applied to convert Ser-36 to Ala in ACP; Primer 14 and primer 15 were applied to convert Lys-122 to His in BCCP87. All mutations were confirmed by DNA sequence analysis.

### *In vitro* modification of ACP and BCCP87

After simultaneous induction by IPTG and arabinose for 4 h, 10 ml of the cell culture harboring pBAD34-A and pCY-A was harvested by centrifugation at 5000 *g* for 10 min and washed with an equal volume of buffer B (50 mM Tris-HCl, pH 8.8). The cells were then resuspended in 5 ml AcpS reaction buffer (50 mM Tris-HCl, pH 8.8, 10 mM MgCl_2_, 5 mM dithiothreitol) and lysed by sonication. The cell lysate was cleared by centrifugation at 12,000 *g* for 20 min at 4°C, 1 mM CoA was added to the supernatant and incubated at 37°C for 4 h [40]. After purification by Ni-chelating affinity chromatography and treatment with TEV protease [41], modified ACP was then subjected to urea-PAGE analysis. Modification of BCCP87 *in vitro* was conducted according to a previous publication [42]. Purified YebF-BCCP87 was incubated with recombinant BirA at 37°C for 3 h in buffer C (3 mM ATP, 5.5 mM MgCl_2_, 60 mM biotin, 100 mM KCl, 5 mM dithiothreitol, and 20 mM sodium phosphate, pH 7.0). EDTA (final concentration 20 mM) was added to samples to stop the reaction. After treatment with TEV protease, modified BCCP87 was then subjected to urea-PAGE analysis.

### Polyacrylamide gel electrophoresis and immunoblotting analysis

Recombinant proteins were first evaluated by 15% SDS-PAGE. ACP and BCCP87 pool compositions were then analyzed by non-denaturing urea-PAGE [27], [43]. Modified, run buffer pH was adjusted to 9.5, recombinant ACP or BCCP87 was mixed with 2×sample buffer containing 1.0 M urea, then applied to a 20% polyacrylamide gel containing 0.5 M urea for a run of approximately 5.0 h, and visualized by staining with Coomassie blue. Images of the gels were analyzed using Quantity One software Version 4.4.0 (Bio-Rad, USA).

After purification with Ni-chelating chromatography and separation by 15% SDS-PAGE, protein fractions were immunoblotted with anti-His_6_ antibody (Novagen). As secondary antibodies, Goat Anti-Mouse IgG and AP conjugate were used for colorimetric detection.

### Mass spectrometry

To verify the modification of ACP by phosphopantetheinylation and BCCP87 by biotinylation, recombinant BCCP87 and ACP were desalted and analyzed by MALDI-TOF-MS (Bruker Autoflex III Smartbean) to determine their molecular masses.

## Supporting Information

Figure S1 **Schematic representation of the recombinant fusion proteins.** (A) fusion protein preYebF-BCCP87. (B) fusion protein YebF-BCCP87. (C) fusion protein preYebF-ACP. (D) fusion protein YebF-ACP. (E) fusion protein preMBP-BCCP87. (F) fusion protein MBP-BCCP87. (G) fusion protein preMBP-ACP. (H) fusion protein MBP-ACP. SP: signal peptide; HT: His_6_-Tag; FP: flexible peptide (ASGGGGA); RS: TEV protease recognition site (ENLYFQ). The calculated molecular weight (MW) of recombinant YebF-BCCP87 is 23.2 kDa; BCCP87 is 9791 Da; YebF-ACP is 22.1 kDa; ACP is 8939 Da.
(TIF)

Figure S2 **SDS-PAGE analysis of the expression of fusion protein Yeb-ACP and Yeb-BCCP87 in** ***E. coli*** **strain JM109 and BL21 (DE3).** After induction with IPTG for 24 h, cells were harvested. Total cell contents (W) were analyzed together with supernatant (S) and periplasm samples (P) after centrifugation and osmotic shock treatment.
(TIF)
