# Supplementary figures and images for: Modified Recombinant Proteins Can Be Exported via the Sec Pathway in Escherichia coli
Source: PLoS One. 2012 Aug 13;7(8):e42519. doi: 10.1371/journal.pone.0042519 (PMC3418276; doi:10.1371/journal.pone.0042519)

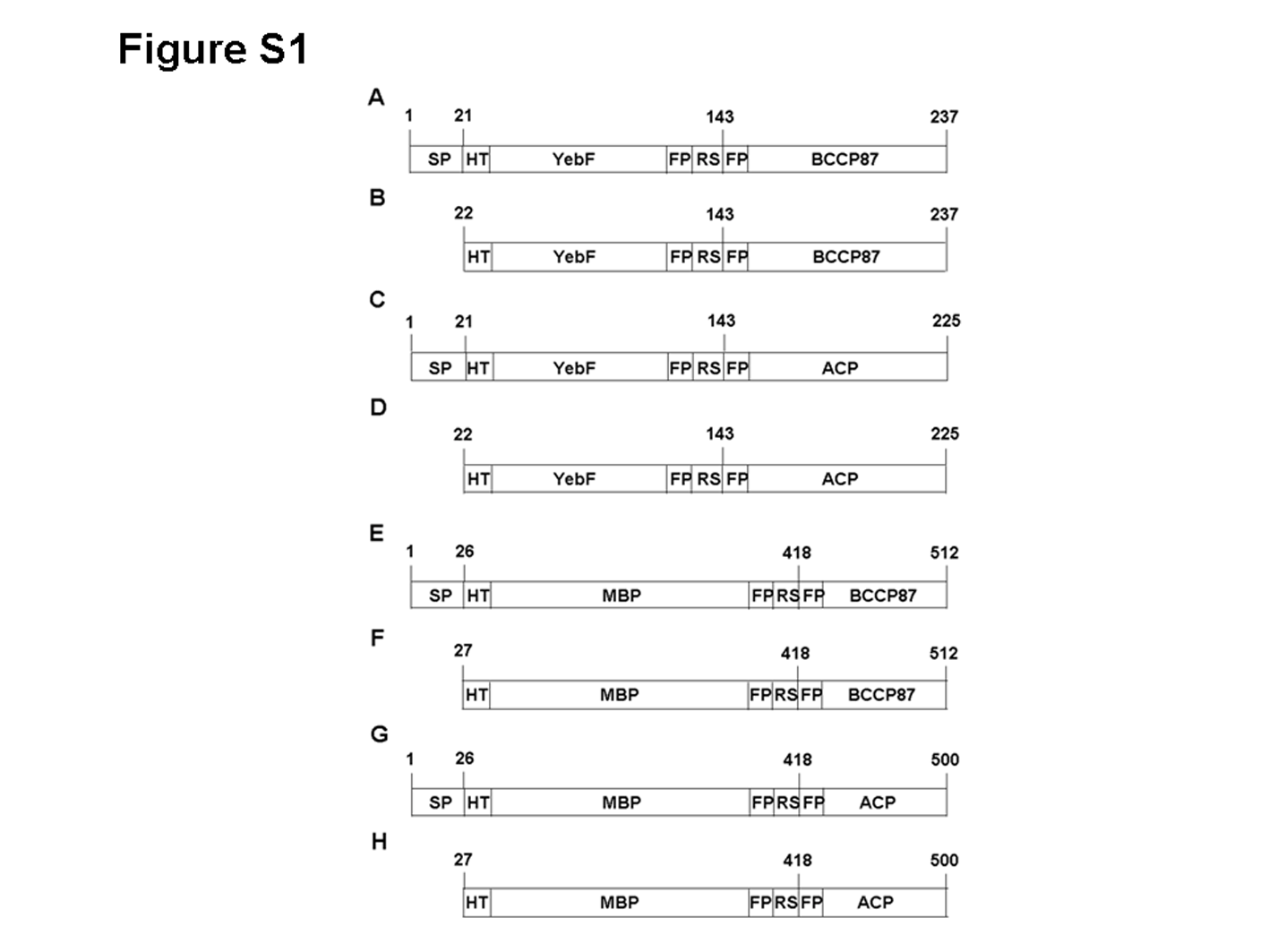

Supplement: Figure S1 — Schematic representation of the recombinant fusion proteins. (A) fusion protein preYebF-BCCP87. (B) fusion protein YebF-BCCP87. (C) fusion protein preYebF-ACP. (D) fusion protein YebF-ACP. (E) fusion protein preMBP-BCCP87. (F) fusion protein MBP-BCCP87. (G) fusion protein preMBP-ACP. (H) fusion protein MBP-ACP. SP: signal peptide; HT: His6-Tag; FP: flexible peptide (ASGGGGA); RS: TEV protease recognition site (ENLYFQ). The calculated molecular weight (MW) of recombinant YebF-BCCP87 is 23.2 kDa; BCCP87 is 9791 Da; YebF-ACP is 22.1 kDa; ACP is 8939 Da. (TIF) [file pone.0042519.s001.tif]

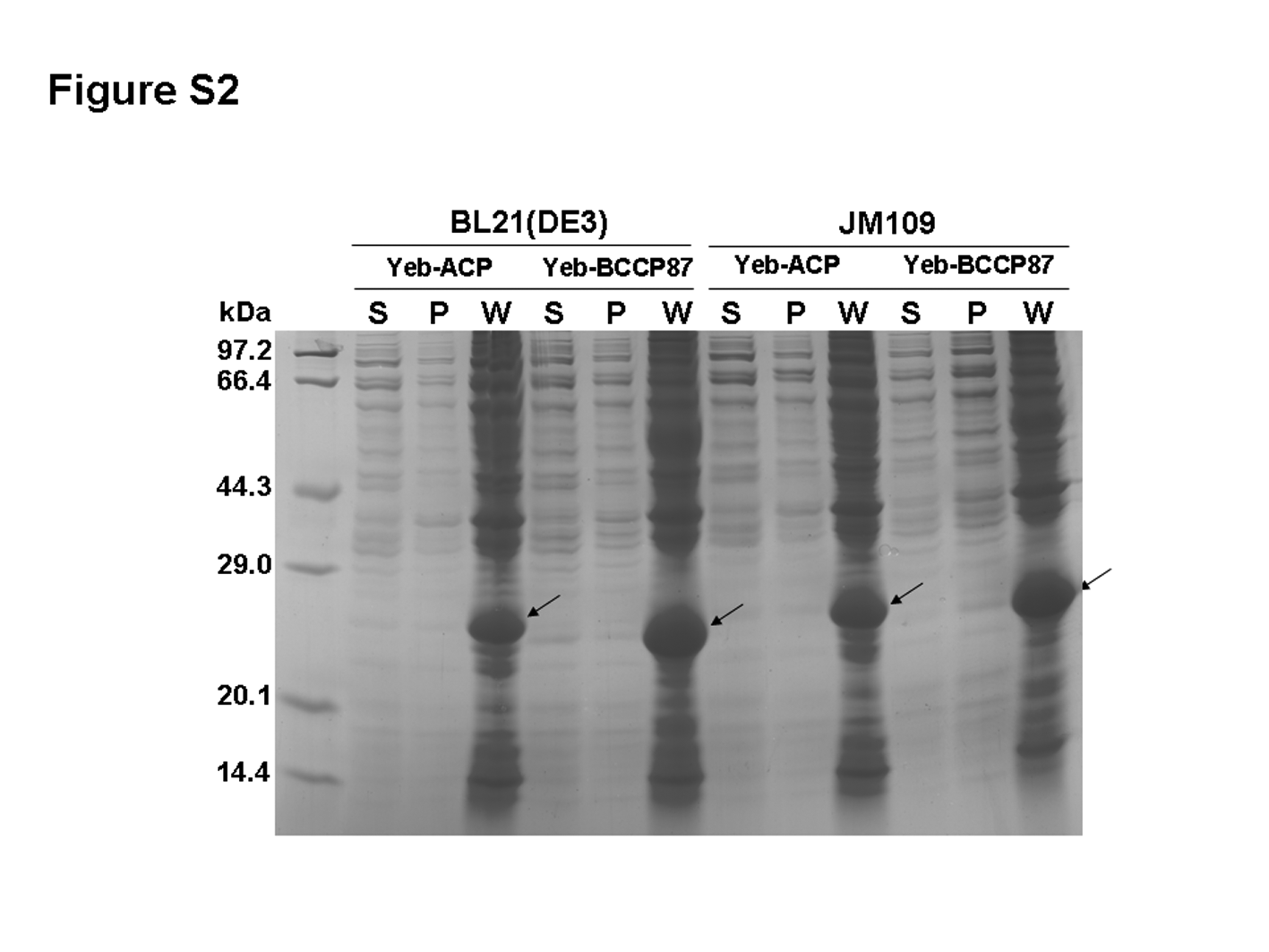

Supplement: Figure S2 — SDS-PAGE analysis of the expression of fusion protein Yeb-ACP and Yeb-BCCP87 in E. coli strain JM109 and BL21 (DE3). After induction with IPTG for 24 h, cells were harvested. Total cell contents (W) were analyzed together with supernatant (S) and periplasm samples (P) after centrifugation and osmotic shock treatment. (TIF) [file pone.0042519.s002.tif]
